# Chitosan-Based Non-viral Gene and Drug Delivery Systems for Brain Cancer

**DOI:** 10.3389/fneur.2020.00740

**Published:** 2020-07-30

**Authors:** Montserrat Lara-Velazquez, Rawan Alkharboosh, Emily S. Norton, Cristopher Ramirez-Loera, William D. Freeman, Hugo Guerrero-Cazares, Antonio J. Forte, Alfredo Quiñones-Hinojosa, Rachel Sarabia-Estrada

**Affiliations:** ^1^Mayo Clinic Florida, Department of Neurosurgery, Jacksonville, FL, United States; ^2^Plan of Combined Studies in Medicine (PECEM), UNAM, Mexico City, Mexico; ^3^Neuroscience Graduate Program, Mayo Clinic Graduate School of Biomedical Sciences, Mayo Clinic, Rochester, MN, United States; ^4^Regenerative Sciences Training Program, Center for Regenerative Medicine, Mayo Clinic, Rochester, MN, United States; ^5^Monterrey Institute of Technology, School of Medicine and Health Sciences, Monterrey, Mexico; ^6^Division of Plastic Surgery and Robert D. and Patricia E. Kern Center for the Science of Health Care Delivery, Mayo Clinic, Jacksonville, FL, United States

**Keywords:** chitosan, brain cancer, brain tumor, nanodelivery, nanoparticles, drug delivery, biodegradable, biomaterials

## Abstract

Central nervous system (CNS) tumors are a leading source of morbidity and mortality worldwide. Today, different strategies have been developed to allow targeted and controlled drug delivery into the brain. Gene therapy is a system based on the modification of patient's cells through the introduction of genetic material to exert a specific action. Administration of the foreign genetic material can be done through viral-mediated delivery or non-viral delivery via physical or mechanical systems. For brain cancer specifically, gene therapy can overcome the actual challenge of blood brain barrier penetration, the main reason for therapeutic failure. Chitosan (CS), a natural based biodegradable polymer obtained from the exoskeleton of crustaceans such as crab, shrimp, and lobster, has been used as a delivery vehicle in several non-viral modification strategies. This cationic polysaccharide is highly suitable for gene delivery mainly due to its chemical properties, its non-toxic nature, its capacity to protect nucleic acids through the formation of complexes with the genetic material, and its ease of degradation in organic environments. Recent evidence supports the use of CS as an alternative gene delivery system for cancer treatment. This review will describe multiple studies highlighting the advantages and challenges of CS-based delivery structures for the treatment of brain tumors. Furthermore, this review will provide insight on the translational potential of various CS based-strategies in current clinical cancer studies. Specifically, CS-based nanostructures including nanocapsules, nanospheres, solid-gel formulations, and nanoemulsions, also microshperes and micelles will be evaluated.

## Introduction

Cancer is the second most frequent cause of mortality following cardiovascular disease, and has surpassed it in high and middle-income countries (1, 2). Although primary malignant central nervous system (CNS) tumors account for 2% of all cancers, they represent a leading cause for morbidity and mortality worldwide. Malignant CNS tumors are the principal cause of death due to solid tumors in children and the third main cause of death in the 15–34 year age-bracket. The most common presentation of a tumor in the brain is due to metastasis, accounting for 40% of intracranial tumors (3). Overall, CNS cancers represent a therapeutic challenge due to tumor heterogeneity, comprised of multiple distinct sup-populations of cells within the same tumor; each with distinct molecular features and biological responses. Furthermore, genetic and epigenetic alterations affect the progression of the disease as well as response to treatment (4). Treatment of brain tumors includes surgical resection; however, due to the infiltrative nature of some tumors, recurrence at original site and surrounding areas (up to 3 cm of the margin of the primary lesion) is often seen, and may be the primary cause of poor prognosis (5). Following surgery, a scheme based on chemotherapy and radiation is considered the standard of treatment. However, limited benefits are achieved after this multimodal strategy, mainly driven by poor drug tissue penetration and accumulation in targeted areas (6).

Nano-delivery systems have been shown to be a promising strategy against multiple types of cancer (7, 8). The possibility of modulating gene expression or the delivery of specific compounds to regulate different pathways in tumor progression has emerged as a promising alternative for CNS malignancies. Drugs or genes are attached to a variety of compounds, followed by a systemic injection or local administration into the tumor (9, 10). Remarkably, the materials show high specificity and tissue penetration in diseased area when injected locally, decreasing systemic toxicity (11, 12). Anatomical barriers, such as the blood brain barrier (BBB), are major challenges to drug penetration, often resulting in therapeutic failure (13). Nano-size materials used as vectors may serve to overcome this limitation and effectively deliver therapeutics to site of injury (9).

In CNS cancers, one of the main challenges is the administration of chemotherapeutic agents and the successful action of the drug in the desired area. For brain tumors, selective penetration of the BBB is a limiting factor for successful eradication of cancer cells (14). Chitosan (CS) -nanomaterials are local delivery systems that overcome the limitations imposed by the BBB, and allow sustained, controlled and prolonged drug release in specific areas, decreasing the risk of systemic toxicity (15). These systems can also be used for real time tracking of cancer cells when acting as imaging probes for various imaging techniques (fluorescent image guiding, magnetic resonance imaging (MRI), computed tomography (CT), positron-emission tomography (PET) and optical imaging) (16). For diagnostic purposes, when compared with free drugs, CS-nanomaterials increase the stability of contrast enhancing agents and drugs with specific accumulation in target areas. Due to their compact size and protein surface interactions, CS-nanostructure components are able to travel in small blood vessels throughout the body. Upon arrival to the tumor area, CS-nanostructures leave the systemic blood flow through disrupted tumor vasculature, and are concentrated and retained in the tumor area (tumor -homing effect) (17).

In this review, we will highlight the recent advances in CS-based gene and drug delivery systems using nanotechnology for the treatment of brain cancer.

## Brain Tumors

Brain tumors are one of the most devastating types of cancer, with the most malignant form having a median survival of ~15 months. Brain tumors can be primary, meaning they arise from the native cells of the brain, or they can be metastatic, arising from tumors that have spread from other organs. Brain tumors have an annual incidence of about 22 people per 100,000 in the United States, with incidence increasing with age (18). Interestingly, brain tumors as a whole occur more frequently in women, while malignant brain tumors are more common in men, indicating a sex difference in brain tumor biology (18). Out of adult primary brain tumors, approximately one-third are malignant (19). Tumors are typically diagnosed through combined neurological exams, MRI of the brain, CT and PET scans to determine whether the tumor is a metastasis arising from another site in the body, and through tumor biopsy (20).

Gliomas, or tumors arising from glial cells, account for over 75% of malignant adult brain tumors. These tumors are classified by the World Health Organization (WHO) by histopathological features and molecular findings. Diffuse gliomas can be stratified by their cell origin through histological characterization (21). The cell of origin is controversial, with various research studies citing neural stem cells as the source of origin, while others cite glial progenitors; classifications are based on features of glial cells. Astrocytomas present features of astrocytes, the star-shaped glial cells important for brain homeostasis, while oligodendrogliomas express features of oligodendrocytes, the cells that produce myelin. Anaplastic astrocytomas and glioblastomas represent 38% of primary brain tumors (3, 22). Diffuse gliomas are classified by the WHO as oligodendrogliomas (grade II), anaplastic oligodendroglioma (grade III), diffuse astrocytoma (grade II), anaplastic astrocytoma (grade III), and the most common glioblastoma (GBM) (grade IV) (21). Gliomas are further defined by their isocitrate dehydrogenase (IDH) 1/2 mutation status. Mutations in IDH 1 and 2 are extremely common in low grade gliomas and secondary high grade gliomas, or high grade tumors that progress from lower grade tumors (23). However, this mutation is relatively rare in primary GBM (23). Additionally, 1p/19q co-deletion, ATRX loss, and TP53 mutation is profiled in order to fully define diffuse gliomas (21). GBM can be further characterized into four molecular subtypes—proneural, neural, classical, and mesenchymal—based on distinct transcriptional signatures (24).

The current standard of care for GBM involves a combinatorial strategy of surgical resection, chemotherapy, and radiation treatment (25). The addition of the chemotherapeutic drug, temozolomide (TMZ), to the treatment strategy in 2005 increased median survival of patients from 12.1 to 14.6, signifying the last major change to GBM treatment (25). Recent medical advances including the development of tumor treating fields via the Optune® system have also shown a significant survival benefit, although these treatments do not provide a cure for GBM (26). The current status of brain tumor management results in a significant need for the development of better therapeutic options to improve patient care (6).

## Non-Viral Mediated Delivery Systems

In contrast to viral vectors, non-viral delivery systems are better tolerated, can carry large amounts of nucleic acid and have a higher safety index due to their transient expression compared to stable modifications (27). CS is an organic molecule that is less toxic than other cationic polymers such as polyethyleneimine, polylysine, or polyarginine (27), and is therefore a promising excipient for non-viral gene and drug delivery systems. Non-viral delivery can be divided into physical or chemical methods (27–30).

### Physical Delivery Systems

Electroporation: An electrical pulse is applied to the cells to increase the permeability of the cell membrane facilitating uptake of DNA strands (31).

Direct injection of nucleic acids: This method has shown a relative degree of success in some tissues, however, without protection following systemic injection, the plasmid DNA (pDNA) is rapidly broken down by nucleases (31).

### Chemical Delivery Systems

Cationic lipids: Lipid-based systems such as FuGene, GenePORTER, Transfast, DOTAP, and Lipofectamine 2000TM are commercially available lipid-based vectors. They are positively charged and encapsulate the anionic nucleic acid to enable cell entry via endocytosis. Lipofectamine 2000TM is the most commonly used reagent and often acts as a positive control in many studies (32).

Cationic polymers: These polymers are positively charged materials that bind electrostatically to negatively charged nucleic acid to form delivery vectors (33). Polymers such as Poly (L-lysine) (PLL), polyethyleneimine (PEI), and Polyamidoamine (PAMAM) dendrimers, have shown promising results pre-clinically, however, toxicity and side effects are often displayed *in vivo* and *in vitro* experiments, ultimately limiting their translational potential (34–36).

Complexation with nucleic acid can reduce the charge of synthetic polymers, for that reason, there is growing concern regarding the degradation and ultimate fate of the construct of non-viral vectors. There is a growing interest in the use of natural biocompatible and biodegradable polymers such as CS (37) which has been used extensively in nucleic acid delivery. CS meets the criteria for a successful non-viral nucleic acid delivery carrier: efficiency in cell uptake, protection of nucleic acids from degradation, efficient unpacking of the genetic cargo, escape from endosomal pathways, and nuclear import (38).

## Biodegradable Polymer: Chitosan

Chitosan is the main derivative of chitin (poly-N-acetyl glucosamine), a linear polysaccharide highly biodegradable and one of the most abundant polymers in nature (second only to cellulose) (11). Partial deacetylation in alkaline conditions of chitin results in the production of CS, a positively charged polysaccharide highly soluble in low pH solutions and poorly soluble in physiological aqueous solutions. CS is present in the exoskeletons of crustaceans (like crabs, lobsters and shellfish), insects and the cellular walls of mycelial fungi with a molecular weight ranging from low (<100 KDa) up to high (>300 KDa) (39). This biomaterial is non-toxic, biocompatible and biodegradable with low allergenicity. It also functions as an antioxidant, hemostatic agent (40, 41); and chelator of elements such as iron, copper and magnesium. CS is cleared by enzymatic hydrolysis mediated by intestinal microorganisms and lysozymes. The main derivatives with medical applicability are N,N,N-trimethyl-CS, N,O-carboxymethyl-CS and O-carboxymethyl-N,N,N-trimethyl-CS (39, 42).

Due to its ability to modulate the inflammatory response, CS has been used for the repair of damaged tissue (wound-healing) by promoting formation of granular tissue after injury (40). Additionally, CS increases the action of neutrophils, macrophages and fibroblasts, ultimately speeding the process of tissue repair. The tissue repair effects of CS are dependent on molecular weight, degree of chemical modification (deacetylation), and CS presentation. Therefore, CS has unique properties that could enhance neuroregeneration by mitigating secondary neuroinflammatory tissue injury. Another strategy for wound healing treatment is through CS-mediated vehicles to deliver growth factors (i.e., FGF, EGF), this option allows for an extended action of the growth factor in the desired location (42).

Antimicrobial action of CS is mediated by its cationic charge that destabilizes the negative bacteria cell membrane, leading to a leakage of inner cellular components (proteins, nucleic acids) and increased permeability in the bacteria cellular membrane impairing nutrient uptake (43). Interestingly, lower concentrations of CS (<0.2 mg/ml) cause bacterial agglutination, while higher concentrations keep them in suspension (40). This biomaterial has broad potency against gram-positive and negative bacteria such as *S. aureus, P. aeruginosa, P. mirabilis*, and *E. Coli* (43); causing osteomyelitis, cystitis, periodontitis, mucositis, burn, and skin infections among others. The potency of CS biomaterial is dependent on the dose, pH and temperature and on the composition of the polymer (hydrogels, coatings, powder, solution, films, pure, or loaded with different materials) (44).

## Chitosan-Based Nanostructures for Brain Cancer Treatment

CS multifunctionality and high cargo entrapment efficiency make CS derivatives versatile nanodelivery vehicles. Chitin monomers are linearized under alkaline conditions by deacetylation in the solid state or by the enzymatic hydrolysis of chitin deacetylases (45). The bipolyaminosaccharide structure is composed of a carbohydrate backbone and abundant –OH and –NH_2_ functional groups that act as readily accessible moieties for functional modifications. This enables tuning for efficient cross-linking, controlled drug release profile, enhanced electrostatic interaction, and increased solubility. The degree of deacetylation and molecular weight ratio of CS-nitrogen to phosphate-cargo make CS a suitable biomaterial that could be utilized for nanoparticle synthesis and nanomaterial fabrication for the delivery of therapeutic agents (46). Amongst the most common nanodelivery systems explored, CS nanoparticles (NPs) have provided a great degree of safety and durability across various pharmaceutical and pre-clinical applications. The inherent cationic nature of CS allows efficient binding to microtubules or motor proteins for cytoplasmic trafficking, increased plasmid or cargo release efficiency mediated by the osmotic pressure in the endosome (caused by influx of hydrogen protons), and finally, low toxicity index due to its biocompatibility and biodegradability across various biological applications (46). Cargo is either complexed or confined inside the CS particle, or dispersed in a CS matrix. These particulate systems can be prepared by cross-linking, cationic salts solvation, emulsification, ionic complexation, or gelation methods by reacting with different functional groups on proteins, antibodies, drugs, DNA/RNA or other pH sensitive moieties (46).

Particles are characterized by their spherical diameter and spatial composition, with **microparticles/microspheres** ranging between 1 and 1,000 μm and **NPs/nanospheres** measuring between 1 nm and >1 μm (47). NPs (and microparticles) are characterized by their constituent components and can be referred to as “**nanocapsules**,” a vesicular particulate system with a hollow sphere consisting of an oil or water core (that may include active cargo), and a polymeric shell (48). This structure mediates cargo entrapment in the core or adsorption on the particle surface. Conversely, matricial structures are referred to as “**nanospheres**” and denote particulate systems where the active molecule is incorporated into the polymer network (48). Moreover, “**solid-lipid**” NPs refer to systems that utilize lipids in the solid phase and subsequent emulsification with a surfactant for structure stability (49). This structure is advantageous when delivering cargo that is poorly water soluble. In line with lipid structures are “**nanoemulsions**” created by the mixture of two immiscible liquids stabilized by a surfactant. Single lipid layer derived particles are referred to as “**micelles**” and do not contain an aqueous core (50). Table 1 summarizes the advantages and disadvantages of the described nanostructures.

**Table 1:** Advantages and disadvantages of distinct nanostructures.

**Morphology**	**Advantages**	**Disadvantages**	**References**
Nanocapsules	Rapid absorption of cargo and longer retention time at target site. Low polymer content required for comparable drug loading. Shell prevents direct contact of cargo with environment offering enhanced protection of load from degradation	Aggregation of particles and leakage of cargo	(11)
Nanospheres	Slow and sustained release of encapsulated cargo. Higher efficiency and low toxicity. More readily protects cargo against reticuloendothelial system	Storage by freezing leads to microfibers. Harsh processing conditions required for scaled-up manufacturing	(51)
Solid-lipid formulations	Versatility of cargo incorporation (hydrophilic and lipophilic) and avoidance of efflux (ex: P-glycoprotein) by exporters on cell membrane	Reorganization of crystalline structure during long storage times could compromise cargo release profile. Low loading efficiency due to “burst effect”	(49)
Nanoemulsions	Oil droplet protect cargo from oxidation and hydrolysis in circulation. Efficient self-assembly and solubilization of lipophilic drugs	Rapid release, low stability and lower encapsulation of hydrophilic molecules	(52)
Micelles	Brain cancer targeting moieties to the vasculature widely studied (transferrin receptor integrins) (53) RGD peptides, LRP1 (LDL Receptor Related Protein 1)	Non-modified micelles display impaired penetration through the BBB (sub-therapeutic delivery of treatment load)	(54)

CS nanosystems are selected based on multiple factors including cargo polarity, solubility, weight, and rout for optimal administration. CS-coated or CS-formulated particulate systems have proven to be efficient nanocarriers to the CNS. Due to their enhanced membrane adhesive nature, particles carrying genes of interest enable enhanced transfection efficiency to recipient cells. Size and composition of CS nanoparticles are fundamental factors that determine targeting and biodistribution to tumors of various origins (55). Nanosized carriers are suitable for disease models that are hypervascularized, such as brain tumors, and would benefit from the enhanced permeability and retention effect permitting passive diffusion in the intratumoral space; this effect limits off-target toxicity (56).

While the development of CS-based nanocarrier technology for brain tumors have primarily focused on the encapsulation and delivery of chemotherapeutics, we will attempt to highlight current advances in non-viral gene delivery strategies using CS nanoparticles along with some promising strategies of drug and chemotherapeutic based encapsulation approaches used for brain cancer treatment.

## Chitosan-Based Delivery-Systems to Brain Cancer

Treatment-resistant brain tumors, such as grade IV gliomas, overexpress epidermal growth factor receptor (EGFR) and galectin-1, leading to chemotherapy resistance. Amplification of EGFR is found in > 50% of GBM cases and presents a logical molecular target for GBM therapy (57). Given the importance of EGFR and its isoforms in brain tumors, several agents have undergone clinical trials in an attempt to target EGFR (i.e., lapatinib, gefitinib) but outcomes have been largely disappointing (58). This is partly due to poor BBB penetration, and the discovery that EGFR receptor blockade is not enough to inhibit downstream signaling, suggesting that EGFR receptor blockade may be activating other pathways that confer cell survival (59). Such a phenomenon would benefit from directed gene silencing. In an effort to examine the silencing efficiency of EGFR and Galectin-1 in U87 human GBM line, CS lipid nanocapsules were complexed with anti-EGFR and anti-galactin-1 small interfering RNA (siRNA) and administered via convection enhanced delivery (CED) (a minimally invasive surgery that placed catheters directly into the tumor bed to deliver pharmaceutical agents) in athymic nude mice (60). Treated groups received concomitant TMZ administration to examine chemotherapy resistance or response after gene silencing. CS nanocapsules carrying EGFR and galectin-1 siRNA significantly increased survival in tumor-bearing mice and decreased gene expression in tumor tissue. While CED administration proved effective in the delivery of CS nanocapsules, another advantage of CS is its mucosal adhesion offering a different administration route. Intranasal delivery has gained momentum in human clinical trials for therapeutic delivery, partly due to its reduced invasiveness and toxicity. CS nanocapsules were delivered intranasally for RNA interference (RNAi) mediated knockdown of galectin-1 in GL261 mouse glioma line, and demonstrated successful nose-to-brain transport of siRNA along with survival benefits when delivered with programmed cell death-1 (PD-1) immunotherapy *in vivo* (61). Data suggests that CS based nanocapsules could effectively translocate across the BBB and deliver nucleic acids to brain cancer *in vitro* and *in vivo* (62, 63).

To circumvent drug delivery limitations to the CNS, poly(lactic-co-glycolic acid) (PLGA) modified CS nanoparticles (CSNPs) were conjugated with Arg-Gly-Asp RGD-linked peptide and loaded with clinically approved paclitaxel (PTX) chemotherapeutic drug for GBM therapy. The PTX-PLGA-CSNP-RGD particle, prepared by emulsion-solvent evaporation, displayed optimal tumor targeting and uptake via integrin receptor mediated endocytosis, induced cell-cycle arrest at G2/M, and increased lung tumor cell death (64). While authors did not address brain tumors in the study, the incorporated RGD linked ligand targets α_v_β_3_ integrins on endothelia and is highly expressed in many tumor vasculature beds but largely absent in normal tissue. Hyper-vascularized tumors, such as brain cancers, would benefit from nano-platforms that incorporate integrin targeting strategies for gene or drug delivery. To bypass limitations of chemotherapeutic delivery to the BBB, dual functionalized liposomes were developed to mitigate transportation of doxorubicin and erlotinib to tumor cells. Liposomes were surface modified with transferrin enabling their translocation across endothelial cells lining the blood vessels, and whose surface exhibits high transferrin receptor expression. Additionally, a cell penetrating PFVYLI peptide was coated on the surface to enhance liposomal uptake by U87 human GBM commercial cell line. GBM cells were seeded in PLGA-chitosan scaffold serving as an *in vitro* porous scaffold 3D platform to study the functional translocation and cellular uptake of coated liposomes. Tumor cells seeded in the PLGA-chitosan scaffold resulted in 52% cell death. This study offers a 3D based platform that acts as a sufficient surrogate to study nanoparticle uptake and translocation in 3D models of brain tumors (65).

Brain targeted chitosan-coated nanoparticles is further shown to enhance particle uptake by human blood-brain barrier cerebral microvessel endothelial cells (hCMECs) via receptor mediated endocytosis. Further evaluation into the mechanisms enabling this translocation revealed a preferential cellular uptake pathway implicating the transferrin receptor with subsequent nanoparticle internalization via receptor-mediated endocytosis (66).

Improved *in vivo* brain pharmacokinetics of conventional GBM chemotherapy, such as TMZ, was shown to be significantly enhanced when polyamidoamine (PAMAM) dendrimer is coated with chitosan and conjugated to TMZ. Chitosan-coated PAMAM conjugated to TMZ improved GBM tumor targeting in U-251 and T-98G cell lines at lower TMZ concentrations. *In vivo* pharmacokinetics exhibited sustained release with a half-life of 22.74 h in chitosan-coated dendrimer compared to free drug (TMZ alone) at 15.35 h. Reported work revealed that chitosan-anchored nanoparticles are sufficient at delivering chemotherapy across the BBB and enhanced tumor cell cytotoxicity *ex vivo* (67).

Similarly, CS nanospheres were constructed by complexing pDNA with CS tripolyphosphate (TPP) and hyaluronic acid (HA) via ionotropic gelation. Ionic gelation permits the formation of sol-gel transition, and TPP stabilizes the complex in biological fluids and decreases particle size (68). Resultant nanosphere (CS-TPP/HA) was evaluated *in vitro* for intracellular delivery of Pseudovirus (pSV)-luciferase (surrogate gene) to neural stem cells and spinal cord slices along with direct injection into the spinal cord *in vivo*. HA signals through CD44 and the receptor for hyaluronan mediated motility (RHAMM) on neural stem cells, regulating proliferation and angiogenesis and mediating the radial migration of spinal cord neurons. CS-TPP/HA resulted in higher gene transfection efficiency, less toxicity, and more retention time of CS nanosphere *in vitro* and *in vivo* compared to PEI or naked-DNA alone, suggesting a viable carrier for gene delivery to neural stem cells using CS nanospheres.

Retinoic acid (RA), a derivative of vitamin A, activates Notch signaling response pathways in glioma initiating stem cells, prompting lineage specific differentiation and arrest at the G0/S phase (69). Strategies to induce cancer stem cell differentiation have been widely used across various malignancies, such as the delivery of bone morphogenetic protein 4 (BMP4) to brain tumor initiating cells for astrocytic induction, rendering cancer stem cells more susceptible to chemotherapy (70). Trimethylated solid-lipid CS formulation was constructed for RA encapsulation to evaluate affinity and delivery of RA to U87 human GBM line (70). N-N-N-trimethyl CS-functionalized (TMC) particles offer increased solubility above native CS solubility threshold (pH <5.6) (71). The polyelectrolytic cationic nature of TMC improves aqueous solubility across a range of pHs while maintaining efficient cell targeting. TMC solid-lipid particles exhibited significant anticancer effects by inducing apoptosis mediated by the delivery of RA, compared to free RA alone. Trimethylated solid-lipid particles offered enhanced protection from the “burst-effect” and prolonged circulation. Modified solid-lipid CS particles hold great promise for cancer therapy as they can deliver sufficient therapeutic pay-loads, entrap hydrophobic drugs at larger concentrations, and improve drug-release profile.

Nanoemulsions are the product of the mixing of two immiscible liquids into a single phase through the use of a surfactant (72). The resulting size of emulsified spheres lies between 10 and 1,000 nm (72). CS has emerged as an attractive coating in nanoemulsions as a way to treat cancer. In particular, CS nanoemulsions have been used to deliver chemotherapeutics in order to increase drug stability, bioavailability of hydrophobic molecules, or drug uptake using positively charged CS to pass the negatively charged biological membranes. In experiments designed to treat brain cancer, the chemotherapeutic 5-fluorouracil (5-FU) has been entrapped into a CS nanoemulsion in order to increase uptake (73). The created nanoemulsion retained more 5-FU within the core matrix of resulting particles, resulting in a slow-release profile from nanoemulsion over a period of 30 days (73). Despite encouraging results on drug release, this study did not perform any results on glioma cell viability. Other studies have found incorporating CS into a 5-FU nanoemulsion increases mucoadhesive properties, contributing to the feasibility of intranasal application (74). Adding CS to the nanoemulsion in this case increased mucoadhesion and resulted in increased drug release *in vivo* in rats (74). Additionally, the 5-FU-loaded CS nanoemulsion resulted in decreased viability and increased apoptosis in C6 rat glioma cells (74), suggesting this may be a practical alternative way to treat glioma. Based on the results found with increased uptake and slow drug release in chemotherapy-loaded CS nanoemulsions, there is high potential for using these methods in DNA-based therapeutics.

Micelles are particles 10–100 nm in diameter formed by amphiphilic molecules self-assembling by turning hydrophobic compartments inward and hydrophilic compartments outward in solution (75). Similar to nanoemulsions, the addition of CS to micelles is appealing as it allows for increased uptake and bioavailability of hydrophobic compounds. Additionally, the loading of therapeutics into micelles with CS may allow for increased transport across the BBB due to the small, amphiphilic nature of the particles. The use of CS micelles in targeting brain cancer has been limited to the use of chemotherapeutics, but has shown to be relatively effective in drug delivery. When CS-containing micelles are loaded with all-trans RA, there is slow drug release and a significant decrease in the migration of U87 GBM cells compared to the application of free drug (76). Similarly, when loaded with a water-insoluble chemotherapeutic, myricetin, there is increased drug uptake, decreased cell viability and increases apoptosis *in vitro* (77). The use of CS micelles with myricetin also decreased tumor growth *in vivo* compared to free drug and controls (77). Adding conjugated CS to micelles can also target glioma cells based on overexpressed receptors on the cell surface. Conjugating CS to d-α-tocopheryl glycol succinate 1000 (TPGS) and incorporating it into docetaxel-loaded micelles allows for targeting to the transferrin receptors on glioma cells (78). This method of targeting glioma cells for chemotherapy is over 200-fold more effective on C6 rat glioma cell viability than free Docel and also exhibits increased cell uptake and stability *in vivo* over time (78). Based on the findings with CS-containing micelles in chemotherapy delivery, this could be a potential future avenue for DNA technology in CNS cancer.

Table 2 summarizes some examples of CS-based formulations used as a cargo for chemotherapeutic delivery against cancer.

**Table 2:** Multiple chitosan applications in cancer drug delivery.

**Disease model**	**Morphology**	**Composition**	**Preparation**	**References**
C6 glioma cells	Nanoemulsions	Polyethylene glycol	Docetaxel loaded D-α-tocopherol polyethylene glycol succinate 1,000 conjugated CS	(79)
RPMI 2,650 human nasal cell line	Nanocapsules	Lipid-core nanocapsules coated with CS	Simvastatin- loaded poly-ε-caprolactone nanocapsules coated with CS	(80)
C6 glioma cells	Nanoemulsions	Oil kaempferol (KPF) (0.1% w/w) in 16% (w/w) medium-chain triglyceride (MCT) and 5.0% (w/w) egg-lecithin	KPF-loaded nanoemulsion and KPF-loaded mucoadhesive nanoemulsion	(74)
GBM	Scaffolds—polyelectrolyte complexes	CS -polyelectrolyte complex scaffolds	Porous CS- scaffolds	(81)
Human brain cancer stem cells	NPs	CS-PLGA NPs modified with sialic acid (SA)	Curcumin -loaded CS- PLGA NPs modified with SA	(82)
T98G human GBM cell line and human umbilical vein endothelial cells	Nanoemulsion	PLGA NPs (50:50)	5-FU PLGA (50:50) NPs, bevacizumab, were loaded into the scaffold	(83)
GBM	Polymeric NPs	Glycol CS and dextran sulfate NPs	Methotrexate—loaded polymeric NPs based on Glycol CS and dextran sulfate	(84)
Human brain cancer cell line (Hs683)	Piperine micellization	Nanomicelles forming core-shell NPs	Optimum piperine-loaded core-shell NPs	(85)
C6 glioma cells	NPs	Glycol CS NPs	MTX-loaded CS NPs	(86)
Mouse fibroblast cell lines L929	NPs	Core–shell polymeric NPs	Docetaxel-loaded NPs	(87)

**Figure 1:**
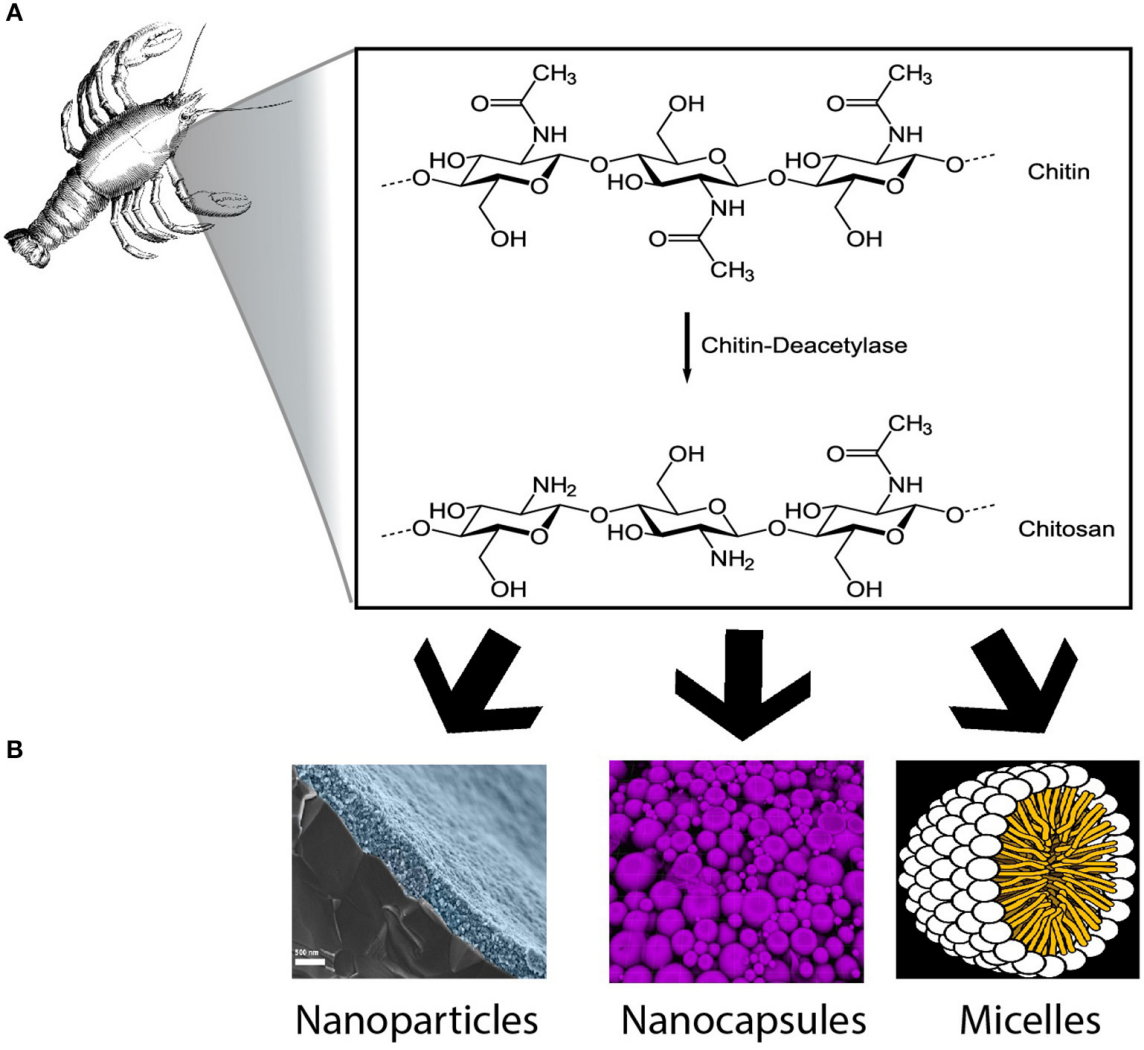
Chitosan based formulations for brain cancer. **(A)** Representation of the chemical conversion from chitin to chitosan. **(B)** Nanoparticles, nanocapsules, and micelles are some examples of chitosan formulations described in this review (Images from **A,B** were obtained from wikimedia commons without any modifications. Chitosan synthesis image by neurotiker, nanoparticles image design by HiguchiJu, nanocapsules image by Stephan Weiss and micelles image by Mariana Ruiz Villareal).

In conclusion, CS has been widely used in several health care materials and extensively studied; chitosan-coated material may offer novel and improved approaches toward the delivery of cancer therapeutics (88). Its biocompatibility and intrinsic characteristics makes it a suitable option to be used as a carrier for brain cancer therapy. Additionally, it is highly available in nature and represents a cost-effective biomaterial for chemotherapeutic delivery to the brain. Based on many pre-clinical studies detailed above, we anticipate that CS will become widely used in upcoming clinical trials and therapeutic development, particularly as a vehicle for previously approved medications and novel DNA gene therapy targeting brain cancer (Figure 1).

## Author Contributions

ML-V, RA, EN, CR-L, and RS-E contributed conception and design of the study. ML-V, RA, EN, and CR-L wrote the first draft of the manuscript and sections of the manuscript. HG-C, AQ-H, WF, and AF contributed reviewing the manuscript. All authors contributed to manuscript revision, read, and approved the submitted version.

## Conflict of Interest

The authors declare that the research was conducted in the absence of any commercial or financial relationships that could be construed as a potential conflict of interest.
